# Extracellular vesicles: pathogenic messengers and potential therapy for neonatal lung diseases

**DOI:** 10.3389/fped.2023.1205882

**Published:** 2023-06-16

**Authors:** Shu Wu, Merline Benny, Joanne Duara, Kevin Williams, April Tan, Augusto Schmidt, Karen C. Young

**Affiliations:** ^1^ Department of Pediatrics, University of Miami Miller School of Medicine, Miami, FL, United States; ^2^ Batchelor Children’s Research Institute, University of Miami Miller School of Medicine, Miami, FL, United States; ^3^ Holtz Children’s Hospital, Jackson Memorial Medical Center, Miami, FL, United States; ^4^ Interdisciplinary Stem Cell Institute, University of Miami Miller School of Medicine, Miami, FL, United States

**Keywords:** extracellular vesicle, neonatal lung disease, bronchopulmonary dysplasia, mesenchymal stem cell (MSC), biomarkers

## Abstract

Extracellular vesicles (EVs) are a heterogeneous group of nano-sized membranous structures increasingly recognized as mediators of intercellular and inter-organ communication. EVs contain a cargo of proteins, lipids and nucleic acids, and their cargo composition is highly dependent on the biological function of the parental cells. Their cargo is protected from the extracellular environment by the phospholipid membrane, thus allowing for safe transport and delivery of their intact cargo to nearby or distant target cells, resulting in modification of the target cell's gene expression, signaling pathways and overall function. The highly selective, sophisticated network through which EVs facilitate cell signaling and modulate cellular processes make studying EVs a major focus of interest in understanding various biological functions and mechanisms of disease. Tracheal aspirate EV-miRNA profiling has been suggested as a potential biomarker for respiratory outcome in preterm infants and there is strong preclinical evidence showing that EVs released from stem cells protect the developing lung from the deleterious effects of hyperoxia and infection. This article will review the role of EVs as pathogenic messengers, biomarkers, and potential therapies for neonatal lung diseases.

## 1. Introduction

Airway cells are often exposed to microbes, environmental insults such as hyperoxia, hypoxia, and mechanical stimuli. These ecological cues induce airway injury, inflammatory responses, and repair processes in the respiratory system. Coordinated intercellular communication is required to maintain lung homeostasis. However, constant exposure to these environmental insults can damage the epithelial barrier leading to excessive inflammatory responses and lung pathology. In the last decade, extracellular vesicles (EVs) have been recognized as important mediators of lung homeostasis and disease (1).

EVs are nano-sized particles characterized based on their physical properties such as size (small EVs are <200 nm and large or medium EVs are >200 nm) or density (low, middle or high), biochemical composition (CD63^+^/CD81^−^ EVs, Annexin A5 EVs, etc.) and description of conditions or cells of origin (lung epithelial cell-derived EVs, podocyte-derived EVs, hypoxia-induced EVs, etc.) (2). EVs contain a cargo of cell-specific lipids, proteins, metabolites, and nucleotides that influence the molecular and functional properties of neighboring and distant target cells (2).

EVs are also categorized based on how they are generated (2). EVs generated by directly budding of the cell plasma membrane have been termed microvesicles, and these are typically 100–1,000 nm in size (3). On the other hand, exosomes (30–100 nm in diameter) are formed from exocytosis of intraluminal vesicles (ILVs). ILVs are generated by endocytosis of cellular cargo (proteins, lipids, metabolites, nucleotides), forming endosomes and subsequently multivesicular bodies (MVBs). MVBs are transported to the plasma membrane through the cytoskeletal and microtubule network. They undergo fusion with the plasma membrane and secretion of ILVs into the extracellular space as exosomes (4). This is regulated by various signaling mechanisms and stimuli, including receptor activation by adenosine triphosphate (ATP) and lipopolysaccharide (LPS) (5, 6). The process also involves the assembly of SNAREs (soluble N-ethylmaleimide-sensitive fusion protein attachment protein receptors) complexes, which draw opposing membranes together to create the energy required for membrane fusion (7). Microvesicles are released through the outward budding and fission of the plasma membrane; this is calcium dependent and associated with cytoskeleton remodeling (3, 8–10).

Specific combinations of proteins and lipids such as tetraspanins, adhesion molecules, glycoproteins, cholesterol, sphingomyelin, and antigen presenting molecules are present on the surface of EVs (2). The exact composition is however dependent on the EV cellular origin, pathogenic conditions, and the mechanism of biogenesis (2). These proteins and lipids influence cellular transport, target cell identification and reception, cargo sorting, and cell programming (8).

**Figure 1:**
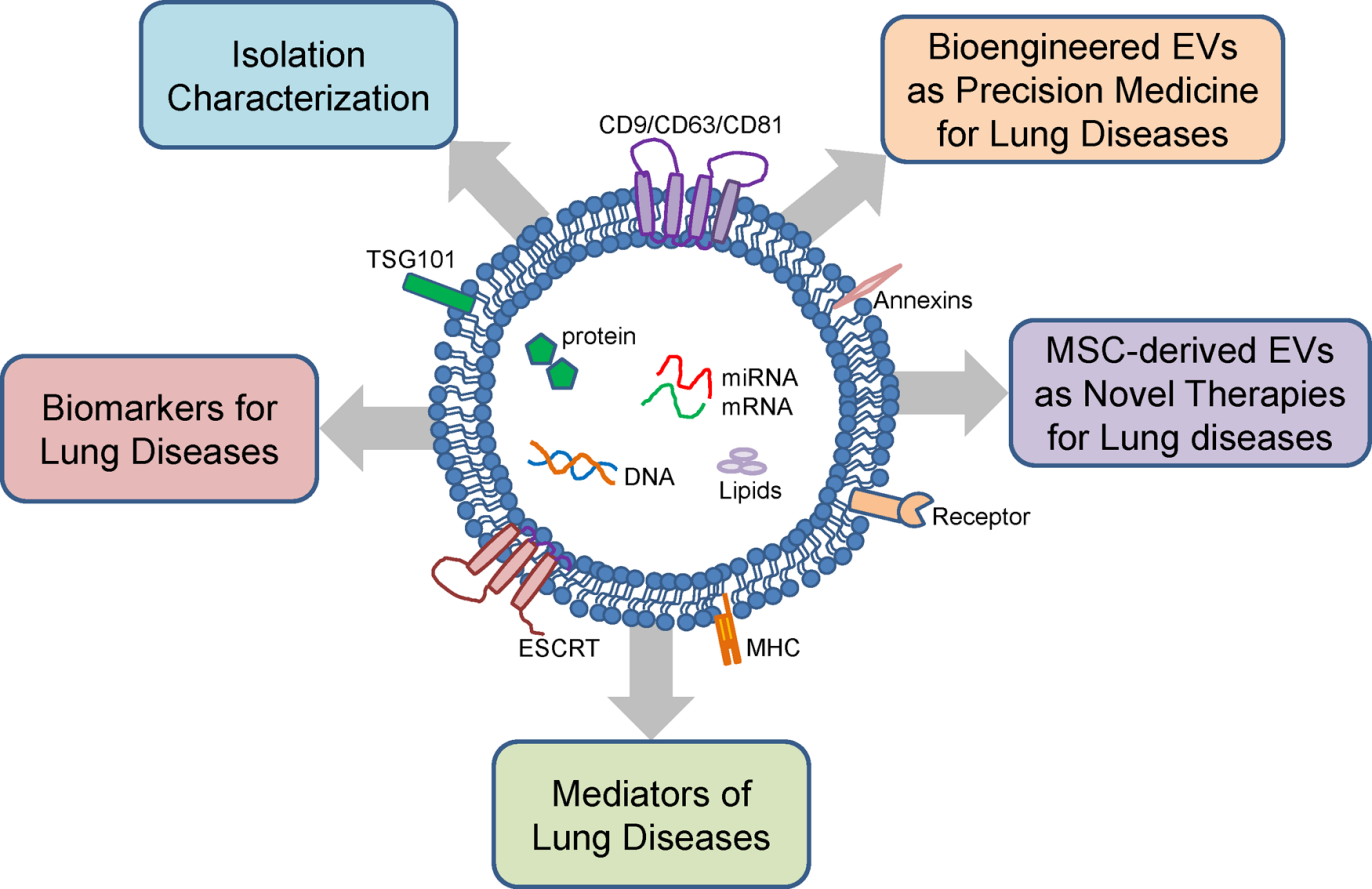
Structure, cargo and function of extracellular vesicles. Extracellular vesicles (EVs) are composed of a lipid bilayer containing transmembrane proteins with cargo consisting of proteins, mRNA, miRNA, DNA, and lipids. EVs can be isolated from various body fluids and have diverse sizes ranging from 100 to 1,000 nm. EVs isolated from the lung fluids and peripheral blood can be used as biomarkers for neonatal lung diseases. EVs have also been linked to the pathogenesis neonatal lung diseases. Mesenchymal stromal cell (MSC)-derived EVs and bioengineered EVs are potential novel therapies for neonatal lung diseases.

EVs are produced by almost all cell types in the respiratory tract (11). Cell types already studied include alveolar type II pneumocytes, pulmonary vascular endothelial cells (PVECs), macrophages, mast cells, and fibroblasts. Under stress such as infection, oxidative stress, and mechanical stress, EVs released by injured lung cells contribute to the development of lung pathologies (12). In addition, lung cell-derived EVs may serve as biomarkers for lung disease risk and severity (11). We will review the mechanisms by which EVs induce lung pathology, the role of EVs as biomarkers in both adult and neonatal lung diseases, and the potential of EVs as vehicles for drug delivery (Figure 1).

## 2. EV isolation

The EV membrane is composed of a phospholipid bilayer containing major histocompatibility complex molecules and tetraspanins. A major challenge of EV research however is achieving high purity EVs while maintaining their integrity and biological activity. Table 1 summarizes common methods of EV isolation.

**Table 1:** Isolation of EVs.

Method	Advantages	Disadvantages	References
Ultracentrifugation	• Gold-standard • Cost-effective • High yield	• Time-consuming • Costly • Easily contaminated • Poor preservation of EV integrity and bioactivity due to high G force	(13, 14)
Ultrafiltration	• Simple • Cheap	• Low-yield • Poor preservation of EV integrity	(15)
Size-exclusion chromatography	• Cheap • Biologically intact EVs • Consistent yield	• Time-consuming but quicker than ultracentrifugation	(15–17)
Polymer precipitation	• Quick, simple process • Cost-effective	• Only smaller volume samples • Extremely prone to contamination by precipitation with non-exosome particles • Inconsistent results	(18, 19)
Immunoaffinity	• Quick, simple process • High purity	• Costly • Only smaller volume samples	(11, 13)
Membrane-based separation	• Quick • High yield • High purity	• Specific sample types only (e.g. urine), unable to handle samples with heterogenous cell types– will affect purity • Membrane clogging	(16)
Microfluidic platforms	• High-throughput • High yield • High purity	• Lack of standardization of devices resulting in heterogenous data • Only smaller volume samples	(20)

Ultracentrifugation is considered the gold standard. It utilizes extremely high centrifugal forces to separate EVs from other biological particles, is affordable and requires little technical skill. However, ultracentrifugation as a purification method is time-consuming, prone to contamination by other particles of similar weight and density, and EV integrity and bioactivity may not be preserved after ultracentrifugation (13, 14, 21).

Ultrafiltration employs physical filters of varying pore sizes and properties like application of electric charge and transmembrane pressure. Ultrafiltration can also be combined with other techniques such as low-speed centrifugation, ultracentrifugation, or size-exclusion chromatography (15). The process of ultrafiltration is simple to perform. However, there are limitations to sample processing – low-yield, membranes clogging, damage to EVs, types of samples that can be ultrafiltered, and the process of ultrafiltration is time-consuming (9).

Size-exclusion chromatography (SEC) is an increasingly popular technique that involves running samples through porous beads leading to separation of molecules by size. EVs isolated by SEC are biologically intact, making this method ideal for functional research (16). When used in conjunction with ultracentrifugation, EV yield and purity are significantly increased (17). The polymer precipitation method utilizes reagents such as polyethylene glycol (PEG) to cause precipitation of EVs, allowing isolation of EVs by simple centrifugation, making it cost-effective and efficient, but prone to contamination (18).

The immunoaffinity technique is done by priming a medium with target antibodies to bind with specific surface antigens or receptors present on EVs of interest. This isolates EVs with high purity, but this method is costly and difficult to sustain. Membrane-based separation methods isolate EVs through binding of membrane hydrophilic phosphate of EVs to metal oxides or the negatively charged membranes to positively charged molecules. This method is high yield, efficient and has high purity rates (19).

Microfluidic platforms are sophisticated networks utilizing various methods of purification organized in a miniature device. Purification methods include immunoaffinity, membrane-based filtration, nanowire trapping, acoustic nanofiltration, deterministic lateral displacement, and viscoelastic flow sorting. Microfluidic devices can achieve high throughput, high yield and high purity EVs, but there is a lack of standardization of devices contributing to heterogeneity of results reported by multiple investigators utilizing various devices (20).

## 3. EV characterization

Analyzing the particle size, morphology and biocomposition of EVs by multiple, complementary techniques is critical in evaluating the likelihood that biomarkers or functions are associated with EVs and not other co-isolated materials (2).

The International Society for Extracellular Vesicles has proposed the Minimal Information for Studies of Extracellular Vesicles-2018 (MISEV2018) guidelines, which recommends that the source and preparation of the EV must be described quantitatively (2). MISEV2018 also recommends using techniques that provide images of single EVs at high resolution such as electron microscopy, using single particle analysis techniques that estimate biophysical features of EVs, and assessing the topology of EV-associated components (2). The commonly used EV characterization techniques are listed in Table 2.

**Table 2:** Characterization of EVs.

Technique	Main features	Drawbacks	References
Nanoparticle tracking analysis (NTA)	• One of the most used methods • Provides parameters of concentration and particle size (10–2,000 nm)	• Critical parameters for success of NTA are sample preparation and the correct dilution factor	(2, 22)
Dynamic light scattering (DLS)	• Used for size measurements in the range of 1–6,000 nm • Possible recovering samples after analysis	• Detection of smaller particles becomes challenging in the mixture of small and large particles	(22, 23)
Atomic force microscopy (AFM)	• Detects the morphology of the sample in three-dimensional space • Generates topographic images of the samples with a resolution limit around 1 nm	• Measures samples in their native condition, which can turn into a limitation of the method as native state of different samples can be varied	(24)
Transmission electron microscopy (TEM)	• Images of high-resolution particles • Using immunogold-labeling to further reveal EV proteins	• Loss of material during extensive sample preparation • Lack of multiparametric phenotyping and low throughput capacity	(25)
Flow cytometry	• Records both the scattering and fluorescence signals • Analyzes multiple labels on individual particles • Identifies various types and subsets	• Low sensitivity to discriminate small size EVs • Low fluorescence being emitted by labeled EVs • Limited feasibility of post-stain washing to reduce background fluorescence	(26)
Protein content of EVs	• Proteomics technology allows the creation of large-scale profiling of proteins secreted through EVs • Immunoblotting can be used to detect EV markers and target proteins	• EVs must be broken prior to analysis • Some of the makers are not present in every/each EV • No single protein or combination of proteins can be recommended as universal EV markers	(27–30)
RNA content of EVs	• High-throughput RNA-seq • Validates by RT-qPCR	• Low yield of materials often below the detection limit of the most common quantification techniques	(27–30)

Dynamic light scattering can be used to measure particle size, but analysis is limited when EVs of various sizes are present, and this cannot be used for functional analysis (22, 23). High resolution flow cytometry is a reliable and popular technique that enables structural analysis, quantification, and functional EV characterization (26). Nanoparticle tracking is a method by which the concentration, size distribution and particle velocity of EVs are measured. In nanoparticle tracking, specific antigens can also be identified with fluorescent tagged antibodies, providing more functional information (22). Atomic force microscopy is a technique that provides outputs of EV quantity, morphology, structural and functional analysis at a molecular level. This technique also preserves the integrity and bioactivity of EVs (24). Electron microscopy (EM) can also be used for structural characterization of EVs. EVs can be visualized with transmission EM, with a characteristic cup-shaped appearance of EVs due to dehydration during sample processing (25). Cryo-EM, on the other hand, allows for visualization of intact EVs without dehydration, enabling ultrastructural analysis of EV membranes and contents (31). EV membrane and cargo components can be analyzed with techniques according to molecule type, such as Western Blot and mass spectroscopy for proteins, and microarray and next generation sequencing for DNA or RNA (27–30).

Given the wide range of techniques available for both isolation and characterization of EVs with varying qualities, and with numerous research studies focusing on EVs that have been reported and are ongoing, there is a need for standardization of research protocols and techniques to maximize knowledge-sharing and productivity of the scientific community. Efforts are being made through the International Society for Extracellular Vesicles (ISEV) to create task forces and research guidelines to overcome these challenges (32, 33).

## 4. EVs in the pathogenesis of lung diseases

Increasing evidence indicates that EVs play essential roles in the pathogenesis of various adult lung diseases, including acute lung injury (ALI), acute respiratory distress syndrome (ARDS), asthma, chronic obstructive pulmonary disease (COPD), and pulmonary hypertension. The involvement of EVs in neonatal lung diseases has also been reported in bronchopulmonary dysplasia (BPD), but much less is known.

### 4.1. EVs and adult lung diseases

ALI and ARDS are devastating and rapidly progressive respiratory disorders that are characterized by disruption of the integrity of alveolar and vascular endothelial barriers (34–36). In response to inflammatory stimuli, EVs and microparticles (MPs) are released from circulating inflammatory cells, damaged PVECs, and epithelial cells (37). In preclinical models, PVEC-derived EVs induce significant lung injury, as demonstrated by alveolar-capillary barrier failure, lung edema, and neutrophil infiltration in mice (38). These pathological effects are linked to and presumably at least in part mediated by the detrimental effects of PVEC-derived EVs on endothelial function. In ALI models, PVEC-derived EVs induce a reduction in endothelial nitric oxide (NO) production and an increased release of lung inflammatory cytokines (39).

Alveolar macrophage derived EVs are also abundant in the bronchoalveolar lavage fluid (BALF) in animal models of ALI. They are capable of inducing inflammatory responses both in vivo and in vitro (37, 40–42). Alveolar macrophage derived EVs trigger EV release by epithelial cells and neutrophils and deliver high concentrations of TNF-α to alveolar epithelial cells, leading to increased production of keratinocyte-derived chemokine and intercellular adhesion molecule-1 (37, 40–42), inducing a vicious cycle of inflammatory injury.

Alveolar epithelial cell derived EVs are also important mediators of ALI. In hyperoxia-induced ALI, alveolar epithelial cell-derived EVs are increased in BALF and serum (43) and they activate proinflammatory responses in systemic and pulmonary macrophages leading to disease progression (44).

COPD is characterized by severe airway inflammation and subsequent lung parenchymal damage. Mononuclear/macrophage-derived EVs rich in inflammatory mediators such as cytokines, chemokines, adhesion molecules, and proteases have been linked to alveolar wall destruction and emphysema, the hallmarks of COPD (11, 45). Endothelial-derived microparticles can promote the progression of COPD by inducing apoptosis of neighboring health endothelial cells upon delivery of inflammatory cargo (46). Epithelial-derived EVs have also been linked to the pathogenesis of COPD. Cigarette smoke stimulates human bronchial epithelial cells to release EVs enriched in full-length CYR61/CTGF/NOV family 1 (CCN1) protein that not only mediates IL-18 induced inflammation but also helps maintain lung homeostasis by increasing the levels of vascular endothelial growth factor (VEGF) (47). Cigarette smoke extract-induced human bronchial epithelial cell-derived EVs promote myofibroblast differentiation of lung fibroblasts, leading to the development of fibrosis (48). Cigarette smoke-exposed lung epithelial cells also release EVs that contain pro-inflammatory cytokines and Wnt-5a into the circulation, and these EVs can reach distant cells and organs (49).

EVs are also implicated in the pathogenesis of pulmonary hypertension. Patients with pulmonary arterial hypertension (PAH) have increased endothelial-derived CD62e microparticles in their pulmonary arterial blood (50). PAH patients also have increased microparticles positive for endothelial PECAM and VE-cadherin in their plasma samples (51). In monocrotaline-induced PAH, lung- and plasma-derived small-sized EVs isolated from monocrotaline-exposed mice induce PAH in healthy mice (52). EVs from PAH mice and patients contain elevated levels of miR-19b, miR-20a, miR-20b, and miR-145, known to target bone morphogenesis protein receptor signaling, apoptosis, and cell proliferation. EVs from the lungs of PAH mice reduce apoptosis of PVECs (53). Furthermore, EVs released by PVECs from PAH mice convert healthy bone marrow-derived endothelial progenitor cells into a pathological progenitor phenotype. These cells induce pulmonary vascular remodeling when injected into the lungs of healthy mice (54).

### 4.2. EVs and bronchopulmonary dysplasia (BPD)

BPD is the most common adverse outcome of extreme prematurity (55). It is the result of antenatal injury to the developing lung combined with repetitive and multiple post-natal insults, including oxygen therapy and ventilation, leading to alveolar simplification and vascular rarefaction (55). Not much is, however, known about the role of EVs in BPD pathogenesis. Genschmer and collaborators compared the function of EVs derived from BALF from BPD and non-BPD infants in a murine model (56). Intriguingly, mice that received intranasal BPD-derived EVs had significant alveolar hypoplasia and right ventricular hypertrophy, suggesting a role for EVs in BPD pathogenesis (56).

Recently, Lal et al. also demonstrated that the tracheal aspirate of infants with severe BPD had higher EV particle concentrations as compared to control infants, and the majority of these EVs were derived from epithelial cells (57). EVs shed from hyperoxia and LPS-exposed epithelial cells had reduced miR-876-3p. Gain of miR-876-3p in murine models attenuated hyperoxia and LPS-induced alveolar simplification, highlighting a potential critical role of lung epithelial cell-derived EV-miRNAs in the pathogenesis of BPD (57). miRNAs are non-coding RNAs that bind to sequences in the 3′ untranslated region (3′UTR) of target mRNA, resulting in the destruction of target mRNA or its repression (58).

Recently, our laboratory investigated the critical role of circulating EVs from hyperoxia-exposed and mechanical ventilated newborn rats in inducing brain injury in healthy newborn rats (59, 60). In the hyperoxia model, newborn rats were exposed to room air or 85% oxygen for two weeks, and circulating EVs were isolated from the plasma of these rats. Fluorescence activated cell sorting (FACS) and Western blot analyses demonstrated that the EVs from hyperoxia-exposed rats contain increased levels of both surfactant C (SPC) and gasdermin D (GSDMD), a key executor of inflammasome-induced cell pyroptosis. When these EVs were adoptively transferred into healthy newborn rats by intra-tail vein injection, they were taken up by the lung and brain. In the lung, the EVs from the hyperoxia-exposed rats induced inflammation, indicated by increased inflammatory cell infiltration in the alveolar airspaces and expression of inflammatory cytokines and chemokines. Furthermore, alveolarization and vascular density were drastically reduced in the lungs that received EVs from hyperoxia-exposed rats. In vitro experiments with PVECs demonstrated reduced cell proliferation and increased cell death when cultured with EVs from hyperoxia-exposed rats (59). Upon examining the brain, EVs from hyperoxia-exposed rats induced brain inflammation by activating microglia and increasing expression of pro-inflammatory cytokines. These changes were associated with increased cell death in the cortex, subventricular zone, and subgranular zone. Additionally, in vitro experiments showed that neural stem cells (NSC) had decreased proliferation and increased cell death when cultured with EVs from hyperoxia-exposed rats (59). EVs from cultured hyperoxia-exposed lung epithelial cells induced pyroptosis in NSC (59). This data revealed a novel lung-brain crosstalk mediated by lung epithelial-derived EVs in both lung and brain injury.

This EV-mediated lung-brain crosstalk was further investigated in mechanical ventilation-associated brain injury in newborn rat models (60). We demonstrated that injurious mechanical ventilation induced similar markers of inflammation and pyroptosis, such as IL-1β and activated caspase-1/GSDMD in both lung and brain, in addition to inducing microglial activation and cell death in the brain (60). EVs isolated from neonatal rats with ventilator-induced lung injury had increased caspase-1. Adoptive transfer of these EVs into healthy newborn rats led to neuroinflammation with microglial activation and activation of caspase-1 and GSDMD in the brain, similar to that observed in neonatal rats that were mechanically ventilated (60). Thus, circulating EVs can contribute to brain injury and possibly poor neurodevelopmental outcomes in preterm infants exposed to hyperoxia and mechanical ventilation (60).

## 5. EVs as biomarkers for lung diseases

The stability of EVs is a potential advantage over traditional biomarkers. Traditional biomarkers such as proteins and RNA molecules are often unstable and susceptible to degradation over time, making them less reliable for diagnostic purposes. In contrast, EVs are surrounded by a protective lipid membrane that helps to stabilize their contents, including proteins, nucleic acids, and other molecular components (61). Proteomic and phosphoproteomic studies conducted on EVs from different cell types have suggested that they transport a diverse range of biologically relevant molecules, such as lipids, carbohydrates, RNAs, and some are believed to exhibit heterogeneity in composition, which is dependent on their cellular origin (62). EVs can carry specific proteins or RNA molecules that are unique to lung diseases. For example, sputum of patients with severe asthma has elevated levels of miR-142-3p, miR-629-3p, and miR-223-3p (63), and sputum-derived EVs from idiopathic pulmonary fibrosis (IPF) patients show an aberrant expression of miR-142-3p, miR-33a-5p, and let-7d-5p compared to healthy subjects (64).

There are few reports that EV-miRNAs can be used as biomarkers for BPD (65). In the study by Lal et al., EV miR876-3p was a potential biomarker for severe BPD in preterm infants. Decreased expression of EV miR-876-3p at birth predicted the future development of severe BPD in ELBW infants (57). This study established the predictive potential and causative role of microbiota-regulated miR-876-3p in severe BPD (57).

More recently, Ransom et al. characterized tracheal aspirate EVs in preterm infants between 22- and 35-week gestational age. Across all gestational ages, the majority of tracheal aspirate EVs expressed epithelial and immune cell markers. Moreover, infants who developed BPD had increased CD14+ EVs in their first tracheal aspirate obtained within 24 h of birth (66).

## 6. EVs as therapies for neonatal lung diseases

Mesenchymal stromal cells (MSCs) have regenerative properties and it is increasingly known that MSC-derived EVs replicate many of the beneficial effects of MSCs. EVs may also be bioengineered for drug delivery and genetically modified to carry specific target molecules. Although these therapeutic strategies are in the early stage of development, the prospect of using them in newborn infants is encouraging.

### 6.1. Stem cell derived EVs for newborn lung diseases

MSCs are efficacious in neonatal lung injury models (67–69). The pleiotropic properties of these cells make them particularly attractive and given their paracrine-mediated mechanism of action, MSC-derived EVs have been investigated as potential therapies.

In an experimental model of chorioamnionitis, antenatal administration of MSC-EVs reduced placental inflammation, and preserved lung structure, suggesting that antenatal MSC-EVs are efficacious in alleviating the deleterious effects of intrauterine inflammation. In experimental pre-eclampsia, MSC-EVs restore placental vascularity and preserve neonatal lung structure (70). In experimental BPD models, MSC-derived EVs restore alveolar structure, prevent lung vascular rarefaction, and alleviate PH by altering macrophage polarization, reprogramming bone marrow myeloid cells and increasing pro-angiogenic signaling pathways (70–77).

We recently compared the therapeutic efficacy of intra-tracheal (IT) and intravenously (IV) delivered MSC-EVs in a preclinical model of BPD. We demonstrated that systemically and IT delivered MSC-EVs have similar beneficial effects in experimental BPD (78). This finding is promising as IV MSC-EVs may also have beneficial effects on the developing brain (79). Another important question which we recently sought to address is the duration of MSC-EV therapeutic effects in experimental BPD. We administered MSC-EVs to neonatal pups with hyperoxia-induced BPD on postnatal day 3 and followed the pups into young adulthood (78). We found that one dose of MSC-EVs at postnatal day 3 had persistent beneficial effects at three month follow up (78). Importantly, late administration of MSC-EVs in an established BPD model was also found to partially reverse lung injury (79, 80). Clinical trials are now on the horizon but identifying the ideal patient will be critical.

### 6.2. Engineered EVs

EVs are also being investigated as “drug vehicles” (81). The ability of EVs to target a particular tissue or cell could be used to deliver drugs to intended targets while avoiding off-targets selectively (81). The “drug cargo” is selectively loaded into the EVs and the EVs are engineered to have specific properties to enhance their targeting and biomimetic features (82, 83). The lower number of transmembrane proteins, such as MHC complexes on their surface, make EVs less immunogenic than their parental source (84, 85). In addition, EVs do not replicate after injection. Thus, EVs are less likely to transfer latent viral pathogens or enable tumor generation (86). Compared to synthetic drug carriers, the intrinsic ability of EVs to cross cell barriers and penetrate tissues gives them an advantage (87). Synthetic drug carriers such as polymeric micelles and lipid nanoparticles cause high toxicity and immunogenicity compared to EVs (88). As therapeutic EVs are derived from benign biological or autologous sources, they are less likely to induce adverse effects.

Harnessing these unique properties of EVs to develop smart drug delivery systems with enhanced targeting, safety and pharmacokinetics has however been challenging (89). One study showed that that after intravenous injection, EVs are rapidly distributed and retained in the liver, spleen, gastrointestinal tract and lungs (90). Another study however showed rapid clearance of plasma-derived EVs following intravenous administration, with a half-life of approximately 7 min (91). Moving forward, more studies will be needed to understand EV circulation kinetics, biodistribution, cell tropism, and intracellular trafficking routes as the cellular origin, dose and route of administration may affect EV biodistribution pattern (92).

Other obstacles such as low isolation yield, the lack of purification protocols, large-scale clinical grade production, parental cell-dependent composition, and inefficient drug payload of the EVs continue to hamper the therapeutic ability of EVs (93). To improve *de novo* EV yield and therapeutic efficacy, re-engineering of the parental cell has been done through genome modification, stimulation with exogenous biomolecules and specific environmental factors (93). Bioreactors are also being extensively used to scale up the production of cell-based therapy and EVs. Bioreactors provide well-controlled nutrients, uniform culture conditions and biomimetic stimuli to regulate cell growth, differentiation and tissue development (94). While bioengineering of the parental cell predictably loads only a small proportion of the modified content into EVs, direct modification of isolated EVs may be another strategy to enrich EVs (95). For example, hydrophobically modified small interfering RNAs efficiently load into EVs upon coincubation, without altering EV size or integrity (96). Active EV loading can also be done by electroporation, sonication, extrusion, freeze-thawing and by surfactant-assisted loading, where surfactant saponin disrupts the membrane and increases its permeability (97).

Another option currently being investigated is the development of artificial EVs, namely the top-down and bottom-up approaches. The top-down approach is based on the disruption of the cultured cells to produce membrane fragments that will be used to form vesicles, while retaining the same membrane features of the initial cell (98). The bottom-up approach starts from small components of molecular building blocks to create complex structures, namely synthetic EVs (99).

## 7. Conclusion

We presented the evidence for lung-derived EVs as novel biomarkers and mediators for neonatal lung diseases and the potential for MSC-derived EVs as novel therapeutic modalities for neonatal lung diseases. Many of the studies discussed in this review are preclinical investigations that require successful translation from the bench to the bedside. Given that lung diseases are among the most common complications in preterm infants, with few effective therapies, it is crucial to continue discovering and understanding how EVs contribute to neonatal lung diseases and how to harness EVs to prevent and treat neonatal lung diseases. The incredible features of EVs in terms of their biocompatibility, cargo loading, cellular uptake, and escaping the immune system make them an appealing therapeutic strategy, but determining the ideal patient, route, dosing and timing will be essential to move forward. Procurement of EVs from physiologically relevant environments, the ability to scale up their manufacturing, optimize their biodistribution, and *in vivo* kinetics will also be crucial (93). This will contribute immensely to increasing the potential of EVs as acellular nanoscale therapeutics for neonatal lung diseases.

## References

[1] Raposo G Stahl PD. Extracellular vesicles: a new communication paradigm? Nat Rev Mol Cell Biol. (2019) 20:509–10. 10.1038/s41580-019-0158-7 31324871

[2] Théry C Witwer KW Aikawa E Alcaraz MJ Anderson JD Andriantsitohaina R Minimal information for studies of extracellular vesicles 2018 (MISEV2018): a position statement of the international society for extracellular vesicles and update of the MISEV2014 guidelines. J Extracell Vesicles. (2018) 7:1535750. 10.1080/20013078.2018.1535750 30637094 PMC6322352

[3] Heijnen HF Schiel AE Fijnheer R Geuze HJ Sixma JJ. Activated platelets release two types of membrane vesicles: microvesicles by surface shedding and exosomes derived from exocytosis of multivesicular bodies and alpha-granules. Blood. (1999) 94:3791–9. 10.1182/blood.V94.11.3791 10572093

[4] Harding C Heuser J Stahl P. Receptor-mediated endocytosis of transferrin and recycling of the transferrin receptor in rat reticulocytes. J Cell Biol. (1983) 97:329–39. 10.1083/jcb.97.2.329 6309857 PMC2112509

[5] Bianco F Pravettoni E Colombo A Schenk U Möller T Matteoli M Astrocyte-derived ATP induces vesicle shedding and IL-1 beta release from microglia. J Immunol. (2005) 174:7268–77. 10.4049/jimmunol.174.11.7268 15905573

[6] Gambim MH de Oliveira do Carmo A Marti L Veríssimo-Filho S Lopes LR Janiszewski M. Platelet-derived exosomes induce endothelial cell apoptosis through peroxynitrite generation: experimental evidence for a novel mechanism of septic vascular dysfunction. Critical Care. (2007) 11:R107. 10.1186/cc6133 17894858 PMC2556756

[7] Fukuda R McNew JA Weber T Parlati F Engel T Nickel W Functional architecture of an intracellular membrane t-SNARE. Nature. (2000) 407:198–202. 10.1038/35025084 11001059

[8] Colombo M Raposo G Théry C. Biogenesis, secretion, and intercellular interactions of exosomes and other extracellular vesicles. Annu Rev Cell Dev Biol. (2014) 30:255–89. 10.1146/annurev-cellbio-101512-122326 25288114

[9] Taylor J Azimi I Monteith G Bebawy M. Ca(2+) mediates extracellular vesicle biogenesis through alternate pathways in malignancy. J Extracell Vesicles. (2020) 9:1734326. 10.1080/20013078.2020.1734326 32194926 PMC7067202

[10] Nabhan JF Hu R Oh RS Cohen SN Lu Q. Formation and release of arrestin domain-containing protein 1-mediated microvesicles (ARMMs) at plasma membrane by recruitment of TSG101 protein. Proc Natl Acad Sci U S A. (2012) 109:4146–51. 10.1073/pnas.1200448109 22315426 PMC3306724

[11] Kubo H. Extracellular vesicles in lung disease. Chest. (2018) 153:210–6. 10.1016/j.chest.2017.06.026 28684288

[12] Kesimer M Scull M Brighton B DeMaria G Burns K O'Neal W Characterization of exosome-like vesicles released from human tracheobronchial ciliated epithelium: a possible role in innate defense. FASEB J. (2009) 23:1858–68. 10.1096/fj.08-119131 19190083 PMC2698655

[13] Coughlan C Bruce KD Burgy O Boyd TD Michel CR Garcia-Perez JE Exosome isolation by ultracentrifugation and precipitation and techniques for downstream analyses. Curr Protoc Cell Biol. (2020) 88:e110. 10.1002/cpcb.110 32633898 PMC8088761

[14] Jeppesen DK Hvam ML Primdahl-Bengtson B Boysen AT Whitehead B Dyrskjot L Comparative analysis of discrete exosome fractions obtained by differential centrifugation. J Extracell Vesicles. (2014) 3:25011. 10.3402/jev.v3.25011 25396408 PMC4224706

[15] Benedikter BJ Bouwman FG Vajen T Heinzmann ACA Grauls G Mariman EC Ultrafiltration combined with size exclusion chromatography efficiently isolates extracellular vesicles from cell culture media for compositional and functional studies. Sci Rep. (2017) 7:15297. 10.1038/s41598-017-15717-7 29127410 PMC5681555

[16] Sidhom K Obi PO Saleem A. A review of exosomal isolation methods: is size exclusion chromatography the best option? Int J Mol Sci. (2020) 21(18):6466. 10.3390/ijms21186466 32899828 PMC7556044

[17] Wei R Zhao L Kong G Liu X Zhu S Zhang S Combination of size-exclusion chromatography and ultracentrifugation improves the proteomic profiling of plasma-derived small extracellular vesicles. Biol Proced Online. (2020) 22:12. 10.1186/s12575-020-00125-5 32587481 PMC7313174

[18] Martinez-Greene JA Hernandez-Ortega K Quiroz-Baez R Resendis-Antonio O Pichardo-Casas I Sinclair DA Quantitative proteomic analysis of extracellular vesicle subgroups isolated by an optimized method combining polymer-based precipitation and size exclusion chromatography. J Extracell Vesicles. (2021) 10:e12087. 10.1002/jev2.12087 33936570 PMC8077108

[19] Deregibus MC Figliolini F D'Antico S Manzini PM Pasquino C De Lena M Charge-based precipitation of extracellular vesicles. Int J Mol Med. (2016) 38:1359–66. 10.3892/ijmm.2016.2759 28025988 PMC5065305

[20] Gao F Jiao F Xia C Zhao Y Ying W Xie Y A novel strategy for facile serum exosome isolation based on specific interactions between phospholipid bilayers and TiO2. Chem Sci. (2019) 10:1579–88. 10.1039/C8SC04197K 30842820 PMC6369439

[21] Linares R Tan S Gounou C Arraud N Brisson AR. High-speed centrifugation induces aggregation of extracellular vesicles. J Extracell Vesicles. (2015) 4:29509. 10.3402/jev.v4.29509 26700615 PMC4689953

[22] Filipe V Hawe A Jiskoot W. Critical evaluation of nanoparticle tracking analysis (NTA) by NanoSight for the measurement of nanoparticles and protein aggregates. Pharm Res. (2010) 27:796–810. 10.1007/s11095-010-0073-2 20204471 PMC2852530

[23] Palmieri V Lucchetti D Gatto I Maiorana A Marcantoni M Maulucci G Dynamic light scattering for the characterization and counting of extracellular vesicles: a powerful noninvasive tool. J Nanopart Res. (2014) 16:2583. 10.1007/s11051-014-2583-z

[24] Sharma S LeClaire M Gimzewski JK. Ascent of atomic force microscopy as a nanoanalytical tool for exosomes and other extracellular vesicles. Nanotechnol. (2018) 29:132001. 10.1088/1361-6528/aaab06 29376505

[25] Rikkert LG Nieuwland R Terstappen L Coumans FAW. Quality of extracellular vesicle images by transmission electron microscopy is operator and protocol dependent. J Extracell Vesicles. (2019) 8:1555419. 10.1080/20013078.2018.1555419 30651939 PMC6327933

[26] Headland SE Jones HR D’Sa AS Perretti M Norling LV. Cutting-edge analysis of extracellular microparticles using ImageStream(X) imaging flow cytometry. Sci Rep. (2014) 4:5237. 10.1038/srep05237 24913598 PMC4050385

[27] Contreras-Naranjo JC Wu HJ Ugaz VM. Microfluidics for exosome isolation and analysis: enabling liquid biopsy for personalized medicine. Lab Chip. (2017) 17:3558–77. 10.1039/C7LC00592J 28832692 PMC5656537

[28] De Sousa KP Rossi I Abdullahi M Ramirez MI Stratton D Inal JM. Isolation and characterization of extracellular vesicles and future directions in diagnosis and therapy. Wiley Interdiscip Rev Nanomed Nanobiotechnol. (2023) 15:e1835. 10.1002/wnan.1835 35898167 PMC10078256

[29] Tiwari S Kumar V Randhawa S Verma SK. Preparation and characterization of extracellular vesicles. Am J Reprod Immunol. (2021) 85:e13367. 10.1111/aji.13367 33118232

[30] Hartjes TA Mytnyk S Jenster GW van Steijn V van Royen ME. Extracellular vesicle quantification and characterization: common methods and emerging approaches. Bioengineering. (2019) 6:7. 10.3390/bioengineering6010007 30654439 PMC6466085

[31] Morandi MI Busko P Ozer-Partuk E Khan S Zarfati G Elbaz-Alon Y Extracellular vesicle fusion visualized by cryo-electron microscopy. PNAS Nexus. (2022) 1:156. 10.1093/pnasnexus/pgac156 PMC9802263 36714848

[32] Royo F Thery C Falcon-Perez JM Nieuwland R Witwer KW. Methods for separation and characterization of extracellular vesicles: results of a worldwide survey performed by the ISEV rigor and standardization subcommittee. Cells. (2020) 9:1955. 10.3390/cells9091955 32854228 PMC7563174

[33] Lai JJ Chau ZL Chen SY Hill JJ Korpany KV Liang NW Exosome processing and characterization approaches for research and technology development. Advanced Science. (2022) 9:e2103222. 10.1002/advs.202103222 35332686 PMC9130923

[34] Mohan A Agarwal S Clauss M Britt NS Dhillon NK. Extracellular vesicles: novel communicators in lung diseases. Respir Res. (2020) 21:175. 10.1186/s12931-020-01423-y 32641036 PMC7341477

[35] Shah T Qin S Vashi M Predescu DN Jeganathan N Bardita C Alk5/Runx1 signaling mediated by extracellular vesicles promotes vascular repair in acute respiratory distress syndrome. Clin Transl Med. (2018) 7:19. 10.1186/s40169-018-0197-2 29931538 PMC6013417

[36] Hu Q Zhang S Yang Y Yao JQ Tang WF Extracellular vesicles in the pathogenesis and treatment of acute lung injury. Mil Med Res. (2022) 9:61. 10.1186/s40779-022-00417-9 36316787 PMC9623953

[37] Mahida RY Matsumoto S Matthay MA. Extracellular vesicles: a new frontier for research in acute respiratory distress syndrome. Am J Respir Cell Mol Biol. (2020) 63:15–24. 10.1165/rcmb.2019-0447TR 32109144 PMC7328246

[38] Buesing KL Densmore JC Kaul S Pritchard KA Jr Jarzembowski JA Gourlay DM Endothelial microparticles induce inflammation in acute lung injury. J Surg Res. (2011) 166:32–9. 10.1016/j.jss.2010.05.036 20828748 PMC4731030

[39] Densmore JC Signorino PR Ou J Hatoum OA Rowe JJ Shi Y Endothelium-derived microparticles induce endothelial dysfunction and acute lung injury. Shock. (2006) 26:464–71. 10.1097/01.shk.0000228791.10550.36 17047516

[40] McVey MJ Maishan M Blokland KEC Bartlett N Kuebler WM. Extracellular vesicles in lung health, disease, and therapy. Am J Physiol Lung Cell Mol Physiol. (2019) 316:L977–89. 10.1152/ajplung.00546.2018 30892076

[41] Li H Meng X Liang X Gao Y Cai S. Administration of microparticles from blood of the lipopolysaccharide-treated rats serves to induce pathologic changes of acute respiratory distress syndrome. Exp Biol Med. (2015) 240:1735–41. 10.1177/1535370215591830 PMC4935343 26088862

[42] Soni S Wilson MR O'Dea KP Yoshida M Katbeh U Alveolar macrophage-derived microvesicles mediate acute lung injury. Thorax. (2016) 71:1020–9. 10.1136/thoraxjnl-2015-208032 27287089 PMC5099194

[43] Moon HG Cao Y Yang J Lee JH Choi HS Jin Y. Lung epithelial cell-derived extracellular vesicles activate macrophage-mediated inflammatory responses via ROCK1 pathway. Cell Death Dis. (2015) 6:e2016. 10.1038/cddis.2015.282 26658190 PMC4720875

[44] Lee H Zhang D Laskin DL Jin Y. Functional evidence of pulmonary extracellular vesicles in infectious and noninfectious lung inflammation. J Immunol. (2018) 201:1500–9. 10.4049/jimmunol.1800264 29997122 PMC6109965

[45] Takahashi T Kubo H. The role of microparticles in chronic obstructive pulmonary disease. Int J Chron Obstruct Pulmon Dis. (2014) 9:303–14. 10.2147/COPD.S38931 24707174 PMC3971913

[46] Letsiou E Bauer N. Endothelial extracellular vesicles in pulmonary function and disease. Curr Top Membr. (2018) 82:197–256. 10.1016/bs.ctm.2018.09.002 30360780 PMC6626636

[47] Moon HG Kim SH Gao J Quan T Qin Z Osorio JC CCN1 secretion and cleavage regulate the lung epithelial cell functions after cigarette smoke. Am J Physiol Lung Cell Mol Physiol. (2014) 307:L326–37. 10.1152/ajplung.00102.2014 24973403 PMC4137167

[48] Fujita Y Araya J Ito S Kobayashi K Kosaka N Yoshioka Y Suppression of autophagy by extracellular vesicles promotes myofibroblast differentiation in COPD pathogenesis. J Extracell Vesicles. (2015) 4:28388. 10.3402/jev.v4.28388 26563733 PMC4643181

[49] Feller D Kun J Ruzsics I Rapp J Sarosi V Kvell K Cigarette smoke-induced pulmonary inflammation becomes systemic by circulating extracellular vesicles containing Wnt5a and inflammatory cytokines. Front Immunol. (2018) 9:1724. 10.3389/fimmu.2018.01724 30090106 PMC6068321

[50] Amabile N Heiss C Chang V Angeli FS Damon L Rame EJ Increased CD62e(+) endothelial microparticle levels predict poor outcome in pulmonary hypertension patients. J Heart Lung Transplant. (2009) 28:1081–6. 10.1016/j.healun.2009.06.005 19782291

[51] Amabile N Heiss C Real WM Minasi P McGlothlin D Rame EJ Circulating endothelial microparticle levels predict hemodynamic severity of pulmonary hypertension. Am J Respir Crit Care Med. (2008) 177:1268–75. 10.1164/rccm.200710-1458OC 18310479

[52] Aliotta JM Pereira M Wen S Dooner MS Del Tatto M Papa E Exosomes induce and reverse monocrotaline-induced pulmonary hypertension in mice. Cardiovasc Res. (2016) 110:319–30. 10.1093/cvr/cvw054 26980205 PMC4872877

[53] Aliotta JM Pereira M Amaral A Sorokina A Igbinoba Z Hasslinger A Induction of pulmonary hypertensive changes by extracellular vesicles from monocrotaline-treated mice. Cardiovasc Res. (2013) 100:354–62. 10.1093/cvr/cvt184 23867631 PMC3826701

[54] Aliotta JM Pereira M Wen S Dooner MS Del Tatto M Papa E Bone marrow endothelial progenitor cells are the cellular mediators of pulmonary hypertension in the murine monocrotaline injury model. Stem Cells Transl Med. (2017) 6:1595–606. 10.1002/sctm.16-0386 28474513 PMC5689760

[55] Thébaud B Goss KN Laughon M Whitsett JA Abman SH Steinhorn RH Bronchopulmonary dysplasia. Nat Rev Dis Primers. (2019) 5:78. 10.1038/s41572-019-0127-7 31727986 PMC6986462

[56] Genschmer KR Russell DW Lal C Szul T Bratcher PE Noerager BD Activated PMN exosomes: pathogenic entities causing matrix destruction and disease in the lung. Cell. (2019) 176:113–26. 10.1016/j.cell.2018.12.002 30633902 PMC6368091

[57] Lal CV Olave N Travers C Rezonzew G Dolma K Simpson A Exosomal microRNA predicts and protects against severe bronchopulmonary dysplasia in extremely premature infants. JCI Insight. (2018) 3:e93994. 10.1172/jci.insight.93994 29515035 PMC5922295

[58] Hu G Drescher K Chen X. Exosomal miRNAs: biological properties and therapeutic potential. Front Genet. (2012) 3:56. 10.3389/fgene.2012.00056 22529849 PMC3330238

[59] Ali A Zambrano R Duncan MR Chen S Luo S Yuan H Hyperoxia-activated circulating extracellular vesicles induce lung and brain injury in neonatal rats. Sci Rep. (2021) 11:8791. 10.1038/s41598-021-87706-w 33888735 PMC8062626

[60] Chavez L Meguro J Chen S de Paiva VN Zambrano R Eterno JM Circulating extracellular vesicles activate the pyroptosis pathway in the brain following ventilation-induced lung injury. J Neuroinflammation. (2021) 18:310. 10.1186/s12974-021-02364-z 34965880 PMC8717639

[61] Sivanantham A Jin Y. Impact of storage conditions on EV integrity/surface markers and cargos. Life. (2022) 12:697. 10.3390/life12050697 35629364 PMC9146501

[62] Guiot J Struman I Louis E Louis R Malaise M Njock MS. Exosomal miRNAs in lung diseases: from biologic function to therapeutic targets. J Clin Med. (2019):8(9):1345. 10.3390/jcm8091345 31470655 PMC6781233

[63] Mortaz E Alipoor SD Varahram M Jamaati H Garssen J Mumby SE Exosomes in severe asthma: update in their roles and potential in therapy. Biomed Res Int. (2018) 2018:2862187. 10.1155/2018/2862187 29854739 PMC5964496

[64] Njock MS Guiot J Henket MA Nivelles O Thiry M Dequiedt F Sputum exosomes: promising biomarkers for idiopathic pulmonary fibrosis. Thorax. (2019) 74:309–12. 10.1136/thoraxjnl-2018-211897 30244194 PMC6467246

[65] Schiller EA Cohen K Lin X El-Khawam R Hanna N. Extracellular vesicle-microRNAs as diagnostic biomarkers in preterm neonates. Int J Mol Sci (2023) 24:2622. 10.3390/ijms24032622 36768944 PMC9916767

[66] Ransom MA Bunn KE Negretti NM Jetter CS Bressman ZJ Sucre JMS Developmental trajectory of extracellular vesicle characteristics from the lungs of preterm infants. Am J Physiol Lung Cell Mol Physiol. (2023) 324:L385–92. 10.1152/ajplung.00389.2022 36719083 PMC10026990

[67] Augustine S Avey MT Harrison B Locke T Ghannad M Moher D Mesenchymal stromal cell therapy in bronchopulmonary dysplasia: systematic review and meta-analysis of preclinical studies. Stem Cells Transl Med. (2017) 6:2079–93. 10.1002/sctm.17-0126 29045045 PMC5702524

[68] Haaften Tv Byrne R Bonnet S Rochefort GY Akabutu J Bouchentouf M Airway delivery of mesenchymal stem cells prevents arrested alveolar growth in neonatal lung injury in rats. Am J Respir Crit Care Med. (2009) 180:1131–42. 10.1164/rccm.200902-0179OC 19713449 PMC3269236

[69] Moreira A Winter C Joy J Winter L Jones M Noronha M Intranasal delivery of human umbilical cord Wharton’s jelly mesenchymal stromal cells restores lung alveolarization and vascularization in experimental bronchopulmonary dysplasia. Stem Cells Transl Med. (2020) 9:221–34. 10.1002/sctm.18-0273 31774626 PMC6988765

[70] Abele AN Taglauer ES Almeda M Wilson N Abikoye A Seedorf GJ Antenatal mesenchymal stromal cell extracellular vesicle treatment preserves lung development in a model of bronchopulmonary dysplasia due to chorioamnionitis. Am J Physiol Lung Cell Mol Physiol. (2022) 322:L179–90. 10.1152/ajplung.00329.2021 34878940 PMC8782653

[71] Taglauer ES Fernandez-Gonzalez A Willis GR Reis M Yeung V Liu X Antenatal mesenchymal stromal cell extracellular vesicle therapy prevents preeclamptic lung injury in mice. Am J Respir Cell Mol Biol. (2022) 66:86–95. 10.1165/rcmb.2021-0307OC 34614384 PMC8803363

[72] Willis GR Reis M Gheinani AH Fernandez-Gonzalez A Taglauer ES Yeung V Extracellular vesicles protect the neonatal lung from hyperoxic injury through the epigenetic and transcriptomic reprogramming of myeloid cells. Am J Respir Crit Care Med. (2021) 204:1418–32. 10.1164/rccm.202102-0329OC 34699335 PMC8865710

[73] Chaubey S Thueson S Ponnalagu D Alam MA Gheorghe CP Aghai Z Early gestational mesenchymal stem cell secretome attenuates experimental bronchopulmonary dysplasia in part via exosome-associated factor TSG-6. Stem Cell Res Ther. (2018) 9:173. 10.1186/s13287-018-0903-4 29941022 PMC6019224

[74] Porzionato A Zaramella P Dedja A Guidolin D Bonadies L Macchi V Intratracheal administration of mesenchymal stem cell-derived extracellular vesicles reduces lung injuries in a chronic rat model of bronchopulmonary dysplasia. Am J Physiol Lung Cell Mol Physiol. (2021) 320:L688–L704. 10.1152/ajplung.00148.2020 33502939

[75] Porzionato A Zaramella P Dedja A Guidolin D Van Wemmel K Macchi V Intratracheal administration of clinical-grade mesenchymal stem cell-derived extracellular vesicles reduces lung injury in a rat model of bronchopulmonary dysplasia. Am J Physiol Lung Cell Mol Physiol. (2019) 316:L6–L19. 10.1152/ajplung.00109.2018 30284924

[76] Ahn SY Park WS Kim YE Sung DK Sung SI Ahn JY Chang YS. Vascular endothelial growth factor mediates the therapeutic efficacy of mesenchymal stem cell-derived extracellular vesicles against neonatal hyperoxic lung injury. Exp Mol Med. (2018) 50:1–12. 10.1038/s12276-018-0055-8 PMC5938045 29650962

[77] Wang J Zhang A Huang F Xu J Zhao M. MSC-EXO and tempol ameliorate bronchopulmonary dysplasia in newborn rats by activating HIF-1α. Pediatr Pulmonol. (2023) 58:1367–79. 10.1002/ppul.26317 36650825

[78] Sharma M Bellio MA Benny M Kulandavelu S Chen P Janjindamai C Mesenchymal stem cell-derived extracellular vesicles prevent experimental bronchopulmonary dysplasia complicated by pulmonary hypertension. Stem Cells Transl Med. (2022) 11:828–40. 10.1093/stcltm/szac041 35758326 PMC9397655

[79] Lithopoulos MA Strueby L O'Reilly M Zhong S Möbius MA Eaton F Pulmonary and neurologic effects of mesenchymal stromal cell extracellular vesicles in a multifactorial lung injury model. Am J Respir Crit Care Med. (2022) 205:1186–201. 10.1164/rccm.202012-4520OC 35286238

[80] Willis GR Fernandez-Gonzalez A Reis M Yeung V Liu X Ericsson M Mesenchymal stromal cell-derived small extracellular vesicles restore lung architecture and improve exercise capacity in a model of neonatal hyperoxia-induced lung injury. J Extracell Vesicles. (2020) 9:1790874. 10.1080/20013078.2020.1790874 32939235 PMC7480622

[81] Reddy SK Ballal AR Shailaja S Seetharam RN Raghu CH Sankhe R Small extracellular vesicle-loaded bevacizumab reduces the frequency of intravitreal injection required for diabetic retinopathy. Theranostics. (2023) 13:2241–55. 10.7150/thno.78426 37153730 PMC10157735

[82] Susa F Limongi T Dumontel B Vighetto V Cauda V. Engineered extracellular vesicles as a reliable tool in cancer nanomedicine. Cancers. (2019) 11(12):1979. 10.3390/cancers11121979 31835327 PMC6966613

[83] Clemmens H Lambert DW. Extracellular vesicles: translational challenges and opportunities. Biochem Soc Trans. (2018) 46:1073–82. 10.1042/BST20180112 30242120

[84] Shigemoto-Kuroda T Oh JY Kim DK Jeong HJ Park SY Lee HJ MSC-derived extracellular vesicles attenuate immune responses in two autoimmune murine models: type 1 diabetes and uveoretinitis. Stem Cell Rep. (2017) 8:1214–25. 10.1016/j.stemcr.2017.04.008 PMC5425726 28494937

[85] Lai RC Tan SS Teh BJ Sze SK Arslan F de Kleijn DP Proteolytic potential of the MSC exosome proteome: implications for an exosome-mediated delivery of therapeutic proteasome. Int J Proteomics. (2012) 2012:971907. 10.1155/2012/971907 22852084 PMC3407643

[86] Murphy DE de Jong OG Brouwer M Wood MJ Lavieu G Schiffelers RM Extracellular vesicle-based therapeutics: natural versus engineered targeting and trafficking. Exp Mol Med. (2019) 51:1–12. 10.1038/s12276-019-0223-5 30872574 PMC6418170

[87] Herrmann IK Wood MJA Fuhrmann G. Extracellular vesicles as a next-generation drug delivery platform. Nat Nanotechnol. (2021) 16:748–59. 10.1038/s41565-021-00931-2 34211166

[88] Witwer KW Wolfram J. Extracellular vesicles versus synthetic nanoparticles for drug delivery. Nat Rev Mater. (2021) 6:103–6. 10.1038/s41578-020-00277-6 36117545 PMC9481198

[89] Gangadaran P Li XJ Lee HW Oh JM Kalimuthu S Rajendran RL A new bioluminescent reporter system to study the biodistribution of systematically injected tumor-derived bioluminescent extracellular vesicles in mice. Oncotarget. (2017) 8:109894–914. 10.18632/oncotarget.22493 29299117 PMC5746352

[90] Wiklander OP Nordin JZ O'Loughlin A Gustafsson Y Corso G Mager I Extracellular vesicle in vivo biodistribution is determined by cell source, route of administration and targeting. J Extracell Vesicles. (2015) 4:26316. 10.3402/jev.v4.26316 25899407 PMC4405624

[91] Matsumoto A Takahashi Y Chang HY Wu YW Yamamoto A Ishihama Y Blood concentrations of small extracellular vesicles are determined by a balance between abundant secretion and rapid clearance. J Extracell Vesicles. (2020) 9:1696517. 10.1080/20013078.2019.1696517 31807238 PMC6882433

[92] Lu M Huang Y. Bioinspired exosome-like therapeutics and delivery nanoplatforms. Biomaterials. (2020) 242:119925. 10.1016/j.biomaterials.2020.119925 32151860

[93] Man K Brunet MY Jones MC Cox SC. Engineered extracellular vesicles: tailored-made nanomaterials for medical applications. Nanomaterials. (2020) 10(9):1838. 10.3390/nano10091838 32942556 PMC7558114

[94] Stephenson M Grayson W. Recent advances in bioreactors for cell-based therapies. F1000Res. (2018) 7:F1000. 10.12688/f1000research.12533.1 29770207 PMC5931275

[95] Didiot MC Hall LM Coles AH Haraszti RA Godinho BM Chase K Exosome-mediated delivery of hydrophobically modified siRNA for huntingtin mRNA silencing. Mol Ther. (2016) 24:1836–47. 10.1038/mt.2016.126 27506293 PMC5112038

[96] Fuhrmann G Serio A Mazo M Nair R Stevens MM. Active loading into extracellular vesicles significantly improves the cellular uptake and photodynamic effect of porphyrins. J Control Release. (2015) 205:35–44. 10.1016/j.jconrel.2014.11.029 25483424

[97] Villata S Canta M Cauda V. EVs and bioengineering: from cellular products to engineered nanomachines. Int J Mol Sci. (2020) 21:6048. 10.3390/ijms21176048 32842627 PMC7504061

[98] Garcia-Manrique P Gutierrez G Blanco-Lopez MC. Fully artificial exosomes: towards new theranostic biomaterials. Trends Biotechnol. (2018) 36:10–4. 10.1016/j.tibtech.2017.10.005 29074309

[99] Minardi S Shah S Luo X. Biomimetic nanoparticles for transplantation tolerance. Curr Opin Organ Transplant. (2018) 23:15–21. 10.1097/MOT.0000000000000485 29140828

